# A streamlined workflow for comprehensive 4DCT imaging QA with automated analysis of spatial integrity and image quality

**DOI:** 10.1002/acm2.70168

**Published:** 2025-07-27

**Authors:** Yunjie Yang, Donghoon Lee, Matthew Whitaker, Wendy Harris, Jeho Jeong, Shih‐Chi Lin, Ellen Yorke, Lakshmi Santanam, Grace Tang

**Affiliations:** ^1^ Department of Medical Physics Memorial Sloan Kettering Cancer Center New York New York USA; ^2^ Image Owl, Inc. Greenwich New York USA

**Keywords:** 4‐dimensional computed tomography, automation, quality assurance

## Abstract

**Purpose:**

A comprehensive 4DCT QA program that includes the assessment of spatial integrity and image quality of 4D phantom scans can be resource‐intensive, especially because the analysis burden scales with the number of motion traces used for QA. This work presents a streamlined and scalable workflow, enabled by the use of a widely available phantom and an automated analysis tool.

**Methods:**

For 4DCT imaging QA, the Catphan was placed on the QUASAR motion platform, driven with sinusoidal motion traces of various amplitudes and frequencies. The acquired image sets were automatically analyzed using a newly designed 4DCT analysis module in TotalQA. The metrics of interest for 4DCT QA were analyzed for image sets of all motion bins and compared to the stationary reference image set. Three broad categories of imaging tests for 4DCT QA were defined: spatial integrity, HU constancy, and image quality. The sensitometry plugs in the Catphan were used as motion surrogates for evaluating spatial integrity, including the dimensions of the plugs and the observed motion amplitude in the scans. HU values of the sensitometry plugs in different phases were evaluated for consistency. For image quality, spatial resolution, low contrast resolution, and image noise were evaluated.

**Results:**

Compared to the stationary reference CT, spatial integrity was within ±1 mm and the HU values were within two HU in the 4DCT for all phases. The spatial resolution was consistent while slightly higher noise was observed in the 4DCT images. The automated analysis in TotalQA was completed in approximately 20 min per motion trace, improving efficiency by more than 80% compared to the manual workflow.

**Conclusions:**

A streamlined 4DCT imaging QA workflow with automated analysis offers a robust assessment of imaging quality and motion accuracy in 4DCT scans, enabling an efficient and consistent QA process in large healthcare networks.

## 1 INTRODUCTION

Respiratory motion can induce large tumor displacement of up to 30 mm in thoracic and abdominal regions, with no discernible correlation between tumor motion and variables such as tumor dimensions, anatomical location, or pulmonary function^1^ Individualized assessment of tumor motion becomes an important consideration for planning and delivery of radiation therapy. A prospective approach to tackle respiratory motion variability in radiation therapy for individual patients involves the utilization of 4‐dimensional computed tomography (4DCT). Analysis of 4DCT data facilitates the determination of the tumor position and its motion range for treatment planning purposes. Utilizing 4DCT enables the delineation of the internal target volume (ITV), while the average CT derived from 4DCT images or free‐breathing CT allows for dose calculation incorporating tumor internal motion.^1^ Therefore, it is crucial to have a comprehensive quality assurance (QA) program to ensure the motion accuracy and image quality of 4DCT are adequate for contouring and dose calculation purposes.

Currently, there is only one set of national guidelines for 4DCT QA. The Canadian Organization of Medical Physicists (COMP) recommends performing 4DCT image quality QA using the Catphan phantom with motion simulation facilitated by the Catphan Shaker.^2^ As Catphan Shaker was discontinued by the manufacturer, different groups have attempted to fill the gap by proposing QA methodologies utilizing motion‐enabled phantoms such as the CIRS Dynamic Thorax Phantom and the QUASAR Respiratory Motion.^3^, ^4^, ^5^, ^6^ However, these studies mainly focused on motion accuracy evaluation and lacked comprehensive image quality tests, as the phantoms used in prior studies did not have dedicated image quality test objects. In addition, the data analysis tools used in these studies were not automated nor commercially available.

In this study, we introduce a streamlined workflow designed to conduct comprehensive 4DCT imaging QA efficiently with a widely available phantom and an automated analysis tool in TotalQA, developed in partnership with Image Owl, Inc. (Greenwich, NY, USA). The automated analysis includes an assessment of both spatial integrity and image quality consistency of each phase of a 4DCT scan. The proposed workflow is almost entirely automated, handling everything from analysis to reporting after images are taken, enabling an efficient, standardized, and scalable process for 4DCT QA.

## 2 MATERIALS AND METHODS

Our institution's 4DCT commissioning and QA procedures were developed based on the COMP report.^1^ In addition to checking the mechanical accuracy (i.e., motion signal acquired by the motion management system), 4D images were assessed for spatial integrity (i.e., dimensions and motion range in different phases of the internal target), HU constancy, and image quality. A series of 4DCT scans were acquired with different motion traces to emulate slow‐ and fast‐breathing patients.

### 2.1 Image quality phantom

The Catphan (The Phantom Laboratory, Greenwich, NY, USA) is a testing instrument for assessing image quality on CT scanners and on‐board imaging systems of linear accelerators (linacs). Its utility extends to monitoring the consistency of CT scanner performance over time, evaluating performance against specifications, or gauging the impact of alterations in clinical scan protocols on image quality.^7^, ^8^ This phantom is widely available and commonly found in radiotherapy departments, often accompanying newly purchased linacs to facilitate quality control testing of on‐board kV imaging systems. Characterized by a modular construction, each module of the Catphan is designed for specific tests, encompassing low contrast resolution, uniformity, sensitometry (i.e., HU constancy), and spatial resolution. In this work, two different models of the Catphan (504 and 604) were used to validate the compatibility of the automated QA software developed.^7^, ^8^

### 2.2 Motion platforms

To simulate motion with the Catphan during CT acquisition, the QUASAR Heavy Duty Respiratory Motion Platform (Modus Medical Devices, London, Canada) was used. The QUASAR motion platform enables programmable 1D motion and can bear weight up to 45 kg. With a relatively large platform area, the Catphan can be safely placed on top of the platform. The motion platform is controlled by dedicated software and allows users to generate a variety of motion traces, including sinusoidal motions as well as importing motion traces. In this study, 1D sinusoidal traces with a 15 mm peak‐to‐peak amplitude with periods of 3, 6, and 10 s were used in addition to a patient motion trace. As proof of principle, 3D patient motion was simulated using a customized 5D HexaMotion motion platform (ScandiDos, Uppsala, Sweden) to demonstrate the compatibility of the 4DCT QA workflow.

### 2.3 Data acquisition

**FIGURE 1:**
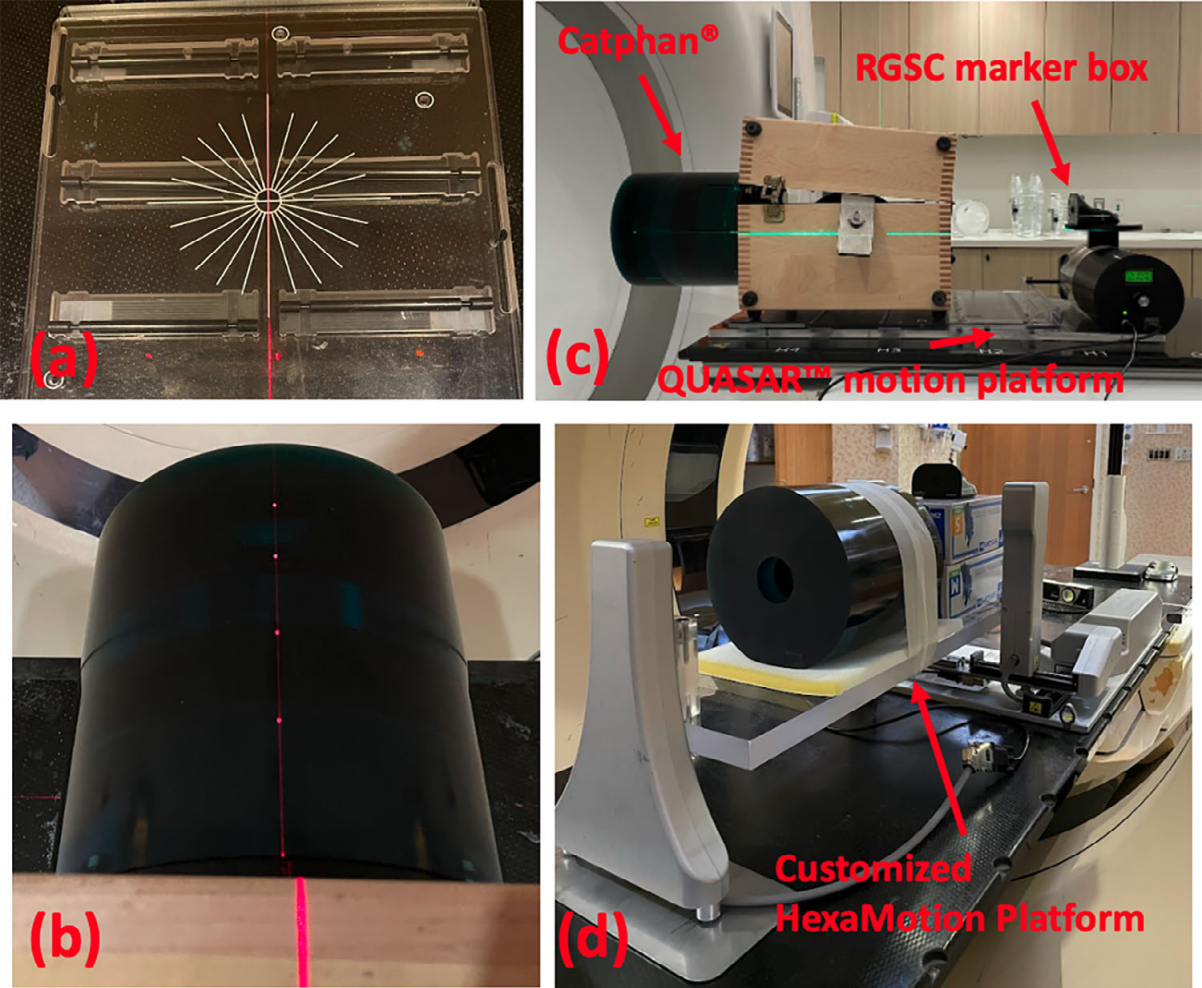
Experimental setup for the 4DCT QA image acquisition: (a) the QUASAR motion platform was first aligned with the room laser, (b) the Catphan was then aligned with the room laser. An overview of the setup is shown in (c). The customized HexaMotion platform with the Catphan is shown in (d).

The 4DCT QA workflow was tested using three CT scanners from three major vendors — Siemens Somatom go.Open Pro, Philips Brilliance Big Bore, and GE Discovery PET/CT. Respiratory signal was provided by the Respiratory Gating for Scanner (RGSC) system (Varian Medical Systems, Palo Alto, CA, USA). Figure 1 illustrates the data acquisition setup of the Catphan on a QUASAR motion platform and on the HexaMotion platform for 4DCT QA scans. The QUASAR platform was first aligned to the in‐room laser system (Figure 1a), followed by positioning the Catphan on the motion platform (Figure 1b). The RGSC marker box was placed on the breathing surrogate platform of the motion platform to provide the motion signals for the RGSC system to perform retrospective‐binning 4DCT image acquisition (Figure 1c). A customized^1^ HexaMotion platform was also used to hold the Catphan and produce 3D motions using a patient trace with the setup shown in Figure 1(d). All scans were performed using our institution's lung imaging protocol with a 2 mm slice thickness. The acquisition conditions for the datasets used in this study are summarized in Table 1.

**TABLE 1:** Imaging tests reported by the automated 4DCT QA analysis module in TotalQA.

Category	Test	Description	Motion trace/platform
Spatial integrity	Motion amplitude	Evaluate the observed range of motion amplitude using the center of a surrogate and compare it to the expected mechanical value of the motion platform input	1D Sinusoidal/QUASAR 1D Patient trace/QUASAR 3D Patient trace/HexaMotion
	Surrogate dimensions	Evaluate the dimensions of the motion surrogates (sensitometry plugs) as seen in 4DCT versus stationary CT	
HU consistency	HU value consistency	Observed HU values of different sensitometry plugs in 4DCT versus stationary	1D Sinusoidal/QUASAR
Image quality	Spatial resolution	MTF at 10% in 4DCT versus stationary CT	1D Sinusoidal/QUASAR
	Low contrast CNR	Low contrast CNR in 4DCT versus stationary CT	
	Noise	Noise metric in 4DCT versus stationary CT	
	Uniformity	Uniformity metric in 4DCT versus stationary CT	

### 2.4 4DCT QA analysis module

In partnership with Image Owl Inc., a new 4DCT imaging QA analysis module was developed in TotalQA, a QA management software. The module was designed to automate the processing and reporting of 4DCT scans of Catphan for QA purposes. In addition to the existing capability of analyzing various image quality metrics such as HU constancy, uniformity, contrast to noise ratio (CNR), and so forth, the newly developed module examines the spatial integrity of the reconstructed 4D images.

The spatial integrity was evaluated in two aspects: (1) the consistency of physical dimensions of the moving target surrogates and (2) the observed motion amplitude in the 4DCT images compared with the expected value. The Teflon and Air plugs of the sensitometry module of the Catphan were chosen as the target surrogates to determine the suitability and robustness for this test due to their high contrast with the surrounding medium. For each Catphan image set, the plugs’ centers were identified, and the HU profiles in the three axes (X/Y/Z) were analyzed. Using a pixel thresholding method, the Teflon and Air plugs were identified. The center of plugs was determined using the centroid of the thresholded region.  Profiles through the centroid were measured to produce the full width at half maximum (FWHM) of the HU profile for each dimension. To assess the size of the moving target in a 4D scan, the average plug dimensions in each of the 10 phases of the 4DCT were computed for all three dimensions. The difference between this average and the corresponding plug dimension measured in the stationary image was extracted. For motion amplitude, the centroids of the target surrogate, that is, the Teflon and Air plugs, were determined in each of the individual phases of the 4DCT scans, and the measured motion amplitude was defined by the extent of motion of these centroid positions in the moving image set. This measured motion amplitude was compared with the input motion trace that drove the motion platform. For the maximum intensity projection (MIP) and minimum intensity projection (MinIP) images, the measured plug dimensions in the z‐direction, that is, the 1D sinusoidal motion direction, were evaluated using the following plug‐projection image pairs: Teflon plug for MIP and Air Plug for MinIP. The expected value would be the sum of the plug z‐dimension in the stationary image and the known input motion platform amplitude.

For image quality, various metrics were evaluated for the stationary scan, each binned image set of the 4D scans, and the average CT calculated from the 4D scans. Geometric distortion was defined as the absolute difference between the average measured distance between spatial targets and the expected distance. Spatial resolution was defined as the line pair per centimeter of the modulation transfer function (MTF) calculated from the upper bead at 10% MTF. The MTF curve is derived from the Fast Fourier Transform of the Point Spread Function of the bead signal. Contrast was defined as the diameter of the smallest detectable object at 1% contrast. HU constancy was defined as the maximum absolute difference from expected CT numbers for air (−1000 HU), LDPE (−100 HU), and acrylic (120 HU) inserts. Uniformity was defined as the maximum absolute difference between four peripheral regions of interest (ROIs) and the center ROI in the uniformity module. Noise was defined as the standard deviation in the measured HU values in the central region of the uniformity module with a diameter of 40% of the total module diameter. The contrast‐to‐noise ratio uses the difference in mean HU values between the polystyrene and LDPE plugs, divided by the norm of the sum of the HU standard deviations of the two plugs. The average values of these metrics of all binned image sets were compared to those of the stationary scan. HU consistency was analyzed similarly, where the HU values of the different plugs in the sensitometry module of the Catphan were analyzed, averaged, and compared to those of the stationary scan. For derived images from 4DCT, including average CT, MIP and MinIP images, the images were analyzed with the same process as the stationary CT. The image quality metrics were analyzed for the average CT, while the apparent plug dimensions were analyzed for the intensity projection images and compared to the expected values.

To evaluate the compatibility of the new 4DCT QA analysis module with 3D motions and with both phase‐based binning and amplitude‐based binning methods, 4DCTs were acquired using a 3D patient motion trace with a customized HexaMotion platform. The 4D scan was reconstructed with both phase‐based binning and amplitude‐based binning, each with 10 phases. The consistency of the plug dimensions was assessed for the two sets of reconstructions.

To validate the accuracy of the QA module, a set of 4DCT images was imported into MIM Maestro (MIM software, Ohio, USA), where spatial integrity was analyzed manually for the Air and Teflon plugs. The contours of both plugs were delineated using HU thresholding using the same FWHM HU value in TotalQA mentioned above. Subsequently, the X/Y/Z dimensions of the plugs were manually measured using the measurement tool and compared with the values obtained by TotalQA. The localization tool provided in MIM was employed to determine the centroid location and to extract the motion amplitude.

## 3 RESULTS

### 3.1 Manual validation

Table 2 shows a three‐way comparison of the plug dimensions and motion amplitude among the reference values (i.e., physical dimensions of the plugs according to the manufacturer's specification and the known physical input motion amplitude), the measurement from the automatic analysis from the newly developed 4DCT QA module in TotalQA, and the measurement from the manual contour‐based analysis performed in MIM. The dataset was acquired from a 4DCT scan with 15 mm peak‐to‐peak amplitude with 6‐second sinusoidal motion. The agreement was within 1 mm for all cases, regardless of the Catphan model (504 or 604), plug type (Teflon or Air), or axis of the plug (X, Y, or Z). These comparisons provided validation of the new 4DCT QA module developed in TotalQA. Some differences were observed in the manual MIM analysis between Catphan 604 versus 504, but this was due to inter‐operator variation, corroborating the importance of a standardized and automated analysis framework.

**TABLE 2:** Comparison of the plug dimensions and motion amplitude with the reference values, the automatic analysis from TotalQA, and the manual analysis performed in MIM.

Comparison of plug dimensions: Reference versus MIM versus TotalQA												
	Catphan 504						Catphan 604					
	Teflon			Air			Teflon			Air		
	X	Y	Z	X	Y	Z	X	Y	Z	X	Y	Z
Nominal Dimensions (mm)	12.3	12.3	25.0	12.3	12.3	25.0	12.3	12.3	25.0	12.3	12.3	33.0
MIM (mm)	12.0 ± 0.1	12.2 ± 0.2	24.0 ± 0.1	11.5 ± 0.1	11.5 ± 0.1	24.0 ± 0.1	12.1 ± 0.2	12.1 ± 0.2	24.1 ± 0.6	12.1 ± 0.2	12.1 ± 0.2	32.4 ± 0.8
TotalQA (mm)	12.2 ± 0.1	12.1 ± 0.1	24.0 ± 0.2	12.0 ± 0.1	12.0 ± 0.1	24.5 ± 0.2	12.1 ± 0.1	12.1 ± 0.1	24.3 ± 0.1	12.1 ± 0.2	12.1 ± 0.1	32.1 ± 0.4

Comparison of motion amplitude: Reference versus MIM versus TotalQA				
	Catphan 504		Catphan 604	
	Teflon	Air	Teflon	Air
Nominal Amplitude (mm)	15.0			
MIM (mm)	15.9	15.8	15.5	15.1
TotalQA (mm)	15.2	15.0	15.1	14.8

### 3.2 Spatial integrity

**FIGURE 2:**
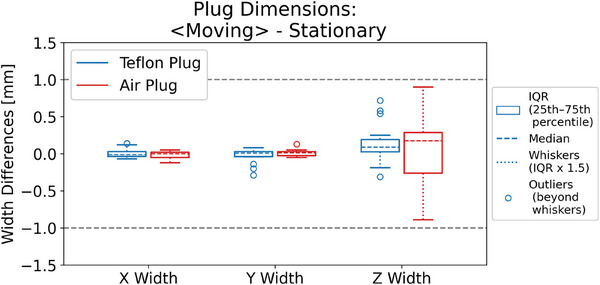
Differences between the average plug dimensions measured in the moving images (i.e., 4DCT) and those measured in the stationary images. The phantom motion was along the Z direction. IQR: inter‐quartile range.

Since of the scope of this study is to present a new 4DCT QA workflow, the vendor/model of the individual CT scanners are blinded in the analysis results. Figure 2 shows box plots of the differences in plug dimensions from 4DCT scans acquired using three motion traces (3, 6, and 10 s periods with 15 mm amplitude) on three CT scanners with two Catphan models, compared with the plug dimensions measured in those of the corresponding stationary images. The agreement between the average plug dimensions measured in the moving images and those measured in the stationary image was within ± 1 mm, as indicated by the dash lines in Figure 2.

**FIGURE 3:**
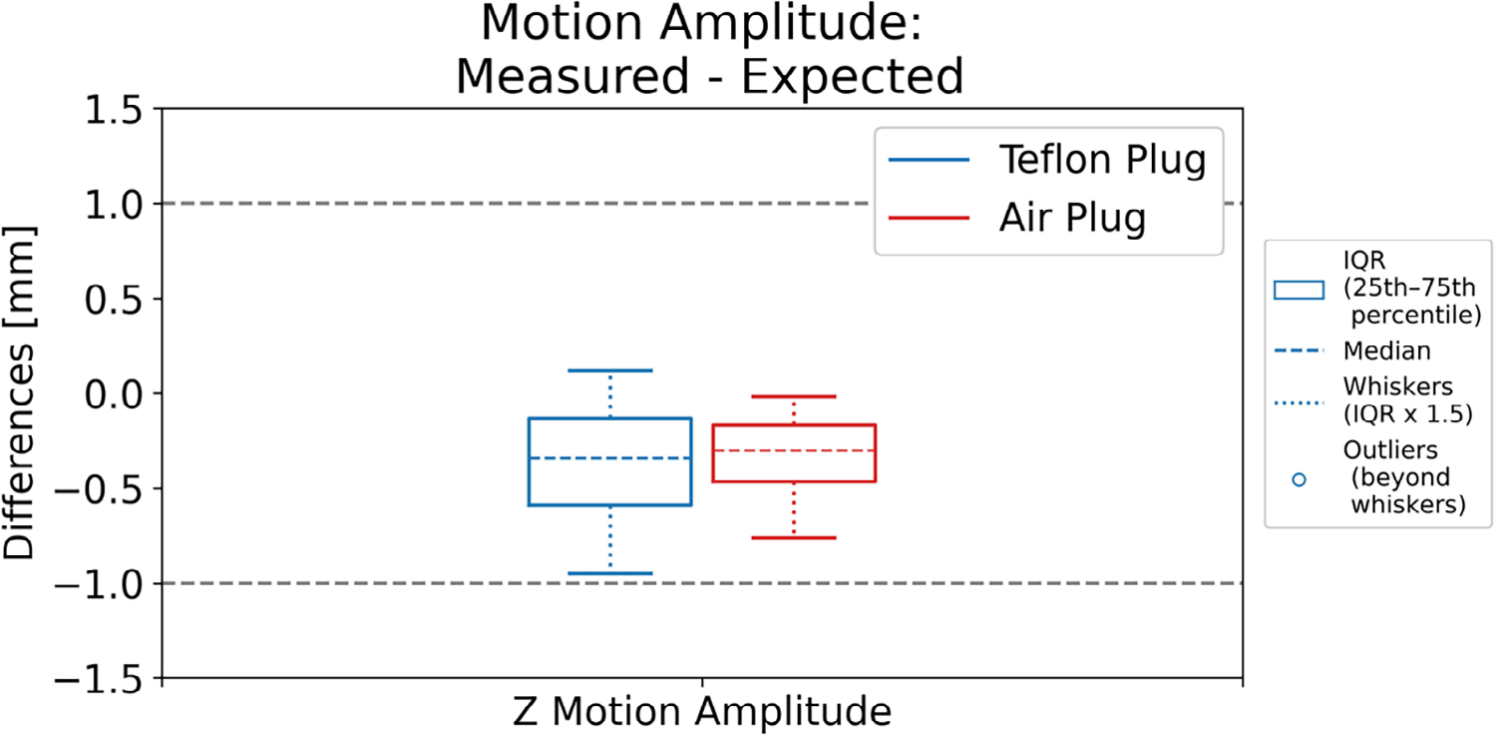
Differences between the measured motion amplitude in the 4DCT moving images and the platform motion amplitude input.

The difference between the measured motion amplitude in the 4DCT moving images and input the platform motion amplitude, acquired with three motion traces on three CT scanners for two Catphan models is shown in Figure 3. The agreement was within ± 1 mm.

**FIGURE 4:**
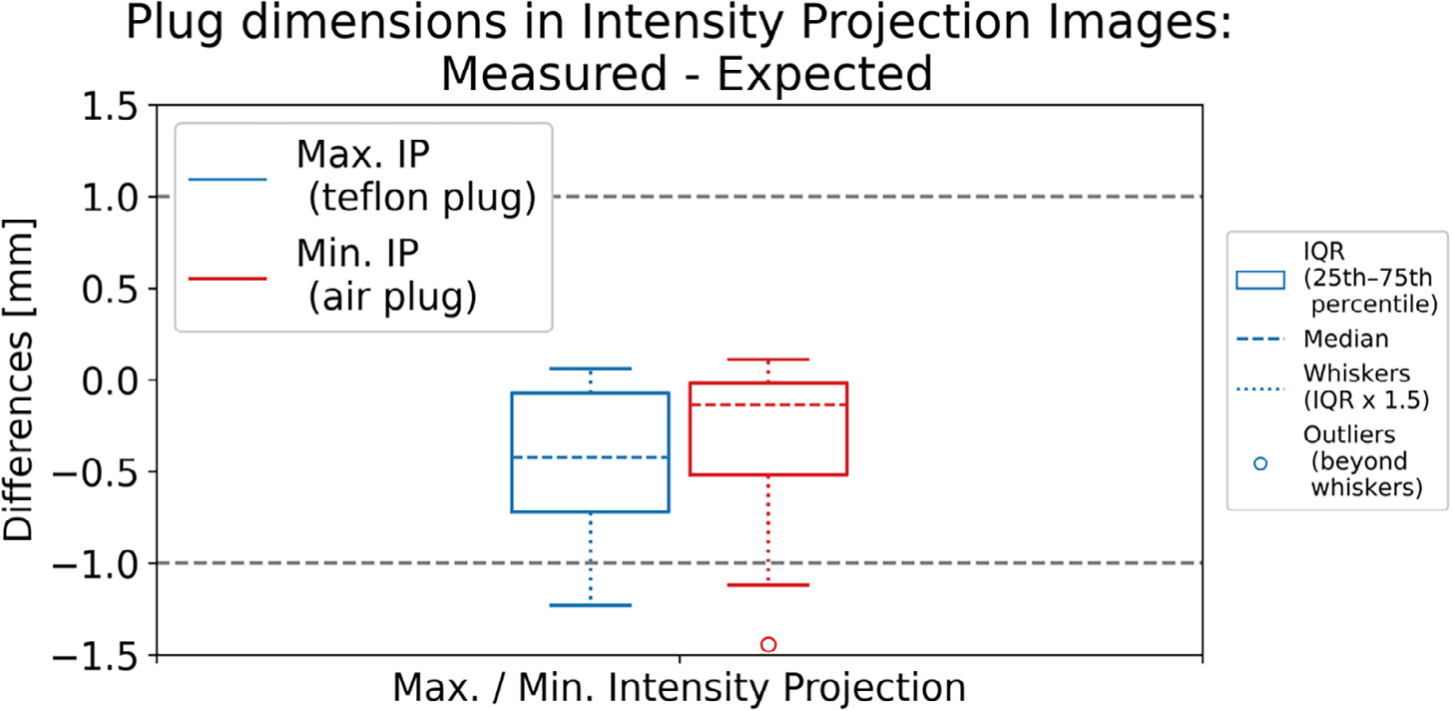
Differences between the measured plug dimensions in the intensity projection images and the expected plug dimensions in these projections.

For the MIP and MinIP images, compared with the expected values, the measured dimensions show about 0.5 mm underestimation from the expected values, but the overall observed spread was still well within ± 1 mm, as shown in Figure 4.

### 3.3 Image quality

Various image quality metrics were evaluated for the individual phases of the 4DCT moving images and the stationary image. The average values were computed for the moving image sets for 4D scans acquired with three motion traces for two Catphan models on three CTs. As shown in Table 3, the 4DCT image quality was largely consistent with that of a standard 3D stationary scan. The image quality metrics of the average CTs were compared with the stationary scan, showing largely consistent results as well. The HU constancy, noise, and low contrast—CNR showed more noticeable differences between the 4DCT and stationary images, which is expected due to differences in the scan and reconstruction protocols.

**TABLE 3:** Comparison of image quality metrics among the moving (4DCT), stationary (Stat.), and the average CT generated from the 4DCT image sets (Avg. CT).

	CT1			CT2			CT3		
	4DCT	Stat.	Avg. CT	4DCT	Stat.	Avg. CT	4DCT	Stat.	Avg. CT
Geom. Dist. [mm]	0.0 ± 0.0	0.0	0.0	0.0 ± 0.0	0.0	0.0	0.0 ± 0.0	0.0	0.2
Spat. Res. [lp/cm]	6.4 ± 0.3	6.5	6.1	6.1 ± 0.2	6.4	5.7	6.3 ± 0.1	6.4	5.9
Contrast	7.7 ± 1.2	5.5	3.0	5.2 ± 0.9	4.0	2.0	4.0 ± 0.1	4.0	1.0
HU constancy [HU]	14.3 ± 2.2	15.8	97.0	35.6 ± 1.7	37.0	71.8	16.0 ± 2.4	7.0	152.2
Uniformity [HU]	3.1 ± 0.9	1.9	3.6	2.6 ± 1.0	2.3	3.8	4.0 ± 1.0	3.8	5.3
Noise [HU]	22.5 ± 0.7	10.9	7.3	14.9 ± 2.8	9.4	4.7	12.4 ± 0.3	12.3	4.7
CNR‐Low Contrast	1.9 ± 0.1	3.8	4.8	2.9 ± 0.5	4.4	6.9	3.7 ± 0.1	3.7	7.9

*Note*: The average values of the image quality metrics are presented alongside with the standard deviation across all binned images for 4DCT.

### 3.4 Compatibility with 3D motion and amplitude binning

**FIGURE 5:**
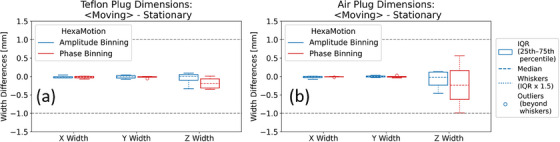
Differences between the average plug dimensions measured in the moving images and those measured in the stationary image using the HexaMotion platform with a 3D representative real patient breathing trace for the Teflon plug (a) and the Air plug (b).

The new 4DCT QA module was able to process and analyze images acquired with 3D motion and was consistent between the two binning methods. Figure 5 shows both phase‐based binning and amplitude‐based binning were able to accurately reconstruct the plug dimensions within ± 1 mm for both Teflon and Air plugs.

### 3.5 Overall efficiency

The analysis portion of the proposed QA workflow is largely automated. Compared to manual analysis, this approach reduces QA time by more than four‐fold and eliminates potential errors inherent in manual analysis. The data acquisition time, including setup, was approximately 30 min for one breathing trace, consisting of about 25 min for equipment and system setup, depending on the user's experience, and 5 min of image acquisition, including scouts, a stationary scan, and the 4DCT acquisition (typically 1–2 min). The images were exported from the scanner and manually imported to TotalQA for analysis. The total time taken to analyze one 4DCT scan was approximately 20 min.

## 4 DISCUSSION

Motion management with 4DCT is prevalent in radiotherapy, yet standardized guidelines for 4DCT QA remain lacking. Currently, COMP provides the only QA guidelines and tolerances, with no similar offerings from their U.S. counterparts. Consequently, clinics often develop their own QA methods, leading to inconsistencies. Additionally, the manual analysis required for 4DCT QA can be time‐consuming, especially in busy environments with multiple CT units.

For equipment selection, the Catphan was found to be the most economical option for our workflow. It is widely accessible and is typically included with new linear accelerator purchases. Although the QUASAR motion phantom and CIRS thorax motion phantom are popular choices for 4D QA applications, they lack comprehensive image quality testing capabilities. On the other hand, the QUASAR motion platform can facilitate motion simulation for dedicated image quality phantoms like the Catphan. Automated Catphan image analysis is a well‐established feature in commercial QA software such as TotalQA, which currently supports Catphan models 504 and 604, with plans to add compatibility for model 503.

In this study, we evaluated motion‐related 4DCT spatial integrity metrics using sensitometry plugs in the Catphan, specifically the Teflon and Air plugs, chosen for their high contrast with surrounding medium. The spatial integrity metrics, including motion target dimensions and amplitude, met expectations within ±1 mm across all motion traces, Catphan models, and CT scanners. However, we observed greater variability in the Air plug dimension metric compared to the Teflon plug in the motion direction, as illustrated in Figures 2 and 5. This variability is expected since dimensions were derived from the FWHM of HU profiles, with the Air plug's lower HU values causing more blurring into adjacent regions due to motion. The plug dimensions measured in intensity projection images showed an approximately 0.5 mm underestimation compared to the expected values from the stationary image and the input motion amplitude. However, the overall spread was still within ±1 mm. The blurring in those derived images likely causes this discrepancy and is under further investigation.

Image quality in 4DCT is greatly influenced by imaging protocols, which can vary between CT scanners and between 4DCT and stationary image sets. Variations in tube energy, mAs, and reconstruction kernels impact the evaluated image quality metrics. In this study, we used the clinical protocols configured at our institution, rather than standardizing across scanners or between 4DCT and stationary acquisitions for the purpose of this study. This approach resulted in differing image quality metrics, as shown in Table 3. In particular, the “noise” and consequently “low contrast—CNR” metrics are expected to be different between the individual bins in a 4DCT scan and the stationary scan due to the different mAs settings. When comparing the average CT with the 4DCT scans and the stationary scans, differences in some image quality metrics, such as the noise and HU constancy, are expected. Because the average CT is generated from the average HU of the individual 4DCT phases, it is expected to have lower noise compared to the individual phase CT and the stationary CT. On the other hand, the averaging operation also applies to the immediate surrounding medium of the sensitometry plugs due to motion, skewing the HU values in those regions. Therefore, the HU constancy metric, which compares the measured with the expected HU values of the sensitometry plugs, is expected to incur larger deviations for the average CT. However, since the primary aim of a QA program is to assess stability and consistency against baseline measurements for the individual CT, the absolute differences observed between CT scanners are not the focus of this study. Our results demonstrate that it is important to establish scanner‐ or protocol‐specific baselines. Nonetheless, the automated analysis in TotalQA will facilitate long‐term monitoring and help establish appropriate tolerance and action limits.

A key strength of this study is the implementation of the new 4DCT QA module in TotalQA, allowing for one‐click automatic analysis of acquired datasets. This substantially reduces the time and effort required from the clinical physicist. The process, including image upload, automatic analysis, and reporting, was conducted without the need of user intervention and completed in under 20 min. Additionally, this study utilized a development version of TotalQA that operated in a single‐threaded manner; the production version will support multi‐threading, further reducing processing time.

In addition to improving efficiency, the 4DCT analysis module in TotalQA also standardizes the analysis process, minimizing user variability. This standardization, along with consistent protocols for phantom selection and data acquisition, enables the collection of homogeneous datasets. These datasets provide clinical physicists with valuable insights into the stability and variability of their equipment through tools like statistical process control, allowing for the establishment of data‐driven tolerance and action limits.^9^ This framework supports prospective risk management tools, such as the failure modes and effects analysis recommended in the AAPM Task Group 100 report, offering a comprehensive view of the 4DCT program's risk profile and optimizing the quality assurance process.^10^

## 5 CONCLUSION

A streamlined 4DCT QA workflow was presented, utilizing the QUASAR motion platform, the Catphan, and a newly developed automatic 4DCT QA analysis module in TotalQA. The analysis module is compatible with two Catphan models and is CT‐vendor agnostic. The proposed workflow takes under 60 min to complete 4DCT QA, from acquisition to analysis.

## AUTHOR CONTRIBUTIONS

All authors contributed to the conception and design, acquisition of data, analysis and interpretation of data, and drafting and revision of the manuscript. All authors reviewed and approved the final version of the manuscript.

## CONFLICT OF INTEREST STATEMENT

Matthew Whitaker is the CEO of Image Owl, Inc.

## Footnotes

1 This platform was customized with a flat platform so that it can hold a variety of phantoms and detectors. The commercially available version is designed specifically for the Delta4 detector (ScandiDos, Uppsala, Sweden).
